# Prior entrepreneurship exposure and work experience as determinants of entrepreneurial intentions among South African university of technology students

**DOI:** 10.3389/fpsyg.2023.1176065

**Published:** 2023-07-13

**Authors:** Mmakgabo Justice Malebana, Simon Thabo Mahlaole

**Affiliations:** Department of Management and Entrepreneurship, Tshwane University of Technology, Pretoria, South Africa

**Keywords:** prior entrepreneurship exposure, prior work experience, entrepreneurial intentions, entrepreneurial knowledge, theory of planned behaviour, South Africa, students

## Abstract

**Purpose:**

This study used the theory of planned behaviour to investigate the effects of prior entrepreneurship exposure and work experience on entrepreneurial intentions among students at a South African university of technology.

**Design/methodology/approach:**

Data was gathered using an online survey questionnaire and convenience sampling. The online survey was completed by 301 entrepreneurship diploma students from the Tshwane University of Technology, and the data were analysed using Smart-PLS 4.

**Findings:**

The findings of this study revealed that prior work experience has a positive and statistically significant relationship with subjective norms and an insignificant relationship with perceived behavioural control, attitude towards behaviour and entrepreneurial intentions. Prior entrepreneurship exposure had a negative but statistically significant relationship with entrepreneurial intentions, subjective norms, attitude towards behaviour and perceived behavioural control. The relationship between prior entrepreneurship exposure and entrepreneurial intentions was partially mediated by subjective norms, attitude towards behaviour and perceived behavioural control. The findings further revealed that perceived behavioural control, subjective norms and attitude towards behaviour had a positive and statistically significant relationship with entrepreneurial intentions.

**Originality:**

The study tested the effects of both prior entrepreneurship exposure and work experience on entrepreneurial intentions and its antecedents which have not been explored fully in previous research. Thus, the study advances the theory of planned behaviour as a model for testing the role of prior entrepreneurship exposure and work experience in the formation of entrepreneurial intentions in the South African context.

## Introduction

The creation and growth of new ventures are vital actions that contribute to economic development and growth and also help to reduce unemployment rate and poverty (Bridge et al., 2009; Storey and Greene, 2010; Shah and Soomro, 2017). The youth in South Africa face high unemployment rates, which stand between 40.5% for those aged 25–34 years and 59.6% for those aged 15–24 years (Statistics South Africa, 2022). These rates challenge both policymakers and higher education institutions to find solutions that would help turn the situation around by encouraging the youth to pursue the entrepreneurial career option and start their own businesses (Lose and Kapondoro, 2020). With the total entrepreneurial activity rate of 2021 standing at 17.5% (Bowmaker-Falconer and Meyer, 2022), South Africa needs more entrepreneurs who could help stimulate the economy and create jobs for the youth who are severely affected by unemployment. However, creating and growing a venture are not responses to stimuli but planned and intentional acts, which are driven by entrepreneurs’ beliefs and perceptions about the attractiveness of the entrepreneurial career option and their capability to act entrepreneurially (Krueger, 1993; Krueger et al., 2000).

Entrepreneurial knowledge is one of the crucial attributes that enhances an individual’s success in launching a new venture that is acquired from prior entrepreneurship exposure (Roxas, 2014; Miralles et al., 2016), and has a positive effect on entrepreneurial intention (Ni and Ye, 2012; Sulistyorini and Santoso, 2021) and the antecedents of entrepreneurial intention (Tshikovhi and Shambare, 2015; Miralles et al., 2016, 2017; Liao et al., 2022). Entrepreneurial knowledge facilitates opportunity recognition, which is the crucial step in the process of creating a new venture (Shane and Venkataraman, 2000; Lim et al., 2023). Therefore, interventions that could assist the youth in acquiring entrepreneurial knowledge and stimulate their entrepreneurial intentions are vital. The significance of these interventions arises from irrefutable theoretical and empirical evidence indicating the pivotal role of entrepreneurial intentions in the new venture creation process (Krueger et al., 2000; Shook et al., 2003).

While Miralles et al. (2017) underscores the importance of direct entrepreneurial experience in equipping individuals with entrepreneurial knowledge, their view appears to discount the value of other forms of prior entrepreneurship exposure which can contribute to the acquisition of entrepreneurial knowledge. According to Bandura (1986), individuals learn through both direct and indirect experiences. Through direct experience with the behaviour, individuals acquire first-hand knowledge about the benefits and challenges of engaging in the behaviour and what it takes to successfully perform the behaviour. Similarly, through observation of role models an individual can indirectly learn about the hardships associated with the behaviour and the positive and negative outcomes that accrue to others as well as the skills, competencies and other attributes that lead to successful performance of the behaviour (Türk et al., 2020). Walter and Dohse (2009) support this argument by indicating that individuals can learn and acquire knowledge from their own direct experiences and from their role models. These forms of prior entrepreneurship exposure can facilitate the acquisition of knowledge relating to the different activities to be undertaken and legal requirements to be followed in creating a new venture (Roxas, 2014; Türk et al., 2020). Since the likelihood of starting a business is linked with confidence in one’s skills and opportunity recognition (Arenius and Minniti, 2005), entrepreneurial knowledge gained through prior entrepreneurship exposure not only can enhance one’s skills but also their ability to recognise opportunities (Davidsson and Honig, 2003; Alnemer, 2021). Additionally, individuals develop intentions to start a business when they have confidence in their own skills and are able to recognise opportunities (Aparicio et al., 2021).

Moreover, entrepreneurial motivation research has shown that negative experiences in the workplaces such as poor pay, not being valued, lack of innovation, limited opportunities to realise one’s full potential, unattractive working conditions, job dissatisfaction, job insecurity, and personal factors could push an individual to pursue the entrepreneurial career option (Henley, 2005; Liang and Dunn, 2006; Wickham, 2006; Hisrich et al., 2008). The entrepreneurial career is reported as having the potential to offer autonomy and intrinsic and extrinsic rewards in various studies, hence it would be perceived as more attractive than a conventional job (Mitchell, 2004; Malebana, 2014a, 2021; Hamilton and de Klerk, 2016). Therefore, with the acquired work experience, it is more likely that individuals would feel confident in their abilities to start their own businesses. However, entrepreneurial intention research has not yet fully accounted for the role of work experience in shaping the formation of entrepreneurial intention and its impact on the antecedents of entrepreneurial intention. Despite the existing research efforts to establish the link between work experience and entrepreneurial intention, these efforts have resulted in mixed findings with some reporting positive, negative or insignificant results (Liñán and Chen, 2009; Walter and Dohse, 2009; Abebe, 2012; García-Rodríguez et al., 2015; Malebana and Swanepoel, 2015; König, 2016; Egerová et al., 2017; Sahinidis et al., 2021). According to Davidsson and Honig (2003), having work experience has a minimal effect on the likelihood of engaging in entrepreneurial activity.

In the light of the above review of prior studies, the purpose of this study is to assess, based on the theory of planned behaviour, the effects of prior entrepreneurship exposure and work experience on entrepreneurial intention and its antecedents. Since knowledge about the determinants of entrepreneurial intentions is vital in shedding light on the precursors of the entrepreneurial behaviour (Krueger et al., 2000), the results of the study have the potential to contribute to the design and implementation of interventions that could stimulate entrepreneurial activity.

This paper is organised as follows: First, we outline the theoretical foundation of this study by discussing the theory of planned behaviour. Second, the role of prior entrepreneurship exposure and work experience in the formation of entrepreneurial intention is outlined, culminating in the formulation of research hypotheses and presentation of the conceptual framework. We then present the methodology that was adopted for this study, followed by reporting of the results. Finally, we highlight the implications and limitations as well as future directions of research.

## Literature review and research hypotheses

### Theory of planned behaviour and entrepreneurial intentions

Entrepreneurial intention that is defined as self-acknowledged conviction by individuals that they intend to establish new business ventures in the future (Thompson, 2009), is the seedbed for entrepreneurial behaviour, whether for new venture creation or growth of existing ventures (Krueger et al., 2000; Shook et al., 2003; Douglas, 2013; Calza et al., 2020; Kong et al., 2020; Akter and Iqbal, 2022; Doung et al., 2022). As a result, over the past three decades researchers have been engaged in the efforts to uncover the factors that influence entrepreneurial intention (Bird, 1988; Krueger, 1993; Krueger et al., 2000; Liñán and Fayolle, 2015). The theory of planned behaviour has emerged as the dominant and popular entrepreneurial intention model for assessing the determinants of entrepreneurial intention (Krueger et al., 2000; Gird and Bagraim, 2008; Liñán and Chen, 2009; Malebana, 2014b; Ebewo et al., 2017; Shah and Soomro, 2017; Mothibi and Malebana, 2019; Urban and Chantson, 2019; Al-Qadasi et al., 2021; Dodescu et al., 2021; Bazan, 2022; Ndofirepi, 2022; Saleh and Manjunath, 2022; Sampene et al., 2022; Zerihun and Edris, 2022). This theory has also demonstrated its relevance in predicting entrepreneurial behaviour (Kautonen et al., 2013; Kibler et al., 2014; Kautonen et al., 2015; Aloulou, 2017; Alam et al., 2019). Findings from some of the recent studies in developing countries indicate that this theory can explain the highest variances of between 77 and 92% in entrepreneurial intention (Tarek, 2017; Sharaf et al., 2018; Urban and Chantson, 2019; Al-Qadasi et al., 2021; Sampene et al., 2022). With regards to entrepreneurial behaviour, studies in other countries have shown that the theory of planned behaviour can account for the variance of between 27 and 39% in behaviour (Kautonen et al., 2013, 2015; Aloulou, 2017).

The theory of planned behaviour suggests that attitude towards behaviour, perceived behavioural control and subjective norms are the primary precursors of intentions (Ajzen, 2005). According to this theory, the formation of entrepreneurial intention is more likely, first, when individuals evaluate the entrepreneurial behaviour and its associated outcomes favourably. Second, individuals should perceive that they have the capability to perform entrepreneurial tasks and can be able to succeed in doing so. Last, individuals should perceive the social pressure to engage in or pursue the entrepreneurial career option. The more individuals value the entrepreneurial behaviour and its outcomes, have confidence in performing entrepreneurial tasks, and perceive the social pressure to engage in entrepreneurship, the stronger will be their entrepreneurial intentions (Ajzen, 2005). Unlike perceived behavioural control and entrepreneurial intention, subjective norms and attitude towards entrepreneurship do not have a direct effect on entrepreneurial behaviour (Ajzen, 2005; Kautonen et al., 2013; Kibler et al., 2014; Kautonen et al., 2015). However, attitudes of potential entrepreneurs shape the start-up vision and influence their creativity in generating strategies for the new venture (Palos-Sanchez et al., 2020). Overwhelming empirical support for the predictive ability of this theory is evident globally, with the results of the majority of previous research indicating the varying effects of the theoretical predictors of entrepreneurial intention (see Mahmoud and Muharam, 2014; Malebana, 2014b; Ndofirepi and Rambe, 2017; Shah and Soomro, 2017; Sharaf et al., 2018; Mothibi and Malebana, 2019; Mahmoud et al., 2020; Dodescu et al., 2021; Bazan, 2022; Sampene et al., 2022). The overwhelming support for this theory suggests that interventions that are directed at changing the antecedents of entrepreneurial intentions and intentions are more likely to bring positive economic results in terms of improved entrepreneurial activity rates and reduced unemployment rates.

### Relationship between prior entrepreneurship exposure, work experience and entrepreneurial intention

New venture creation represents an important aspect of entrepreneurship, which is ‘agent-dependent’ (Shane and Venkataraman, 2000; Davidsson, 2021). To successfully start and effectively manage a new venture, entrepreneurs as agents in the entrepreneurial process require a variety of entrepreneurial skills (Kickul and D’Intino, 2005) and competencies (Nabila and Ambad, 2022). While these skills and competencies are vital in the identification, evaluation and exploitation of opportunities (Wickham, 2006), they also contribute immensely in the creation, survival and growth of a new venture (Arenius and Minniti, 2005; Khoshmaram et al., 2018). Since the formation of entrepreneurial intention and the likelihood of becoming a nascent entrepreneur are positively associated with one’s ability to recognise opportunities (Arenius and Minniti, 2005; Anwar et al., 2021), prior entrepreneurship exposure and work experience can become the means through which individuals can gain prior information that facilitates opportunity recognition and for enhancing individuals’ necessary cognitive properties to value the identified opportunities (Shane and Venkataraman, 2000).

Prior entrepreneurship exposure and work experience can equip an individual with vital knowledge about the markets, customer problems and needs and how to serve customers, which promote alertness to market opportunities (Ardichvili et al., 2003; Tang, 2008). Depending on the nature of opportunities to be identified and exploited, an individual’s prior industry experience and entrepreneurship exposure in related business activities can facilitate successful opportunity identification and exploitation (Moreno, 2008; Smith et al., 2009). Furthermore, once the venture has been created, entrepreneurs can capitalise on their prior experience to ensure venture survival (Linder et al., 2020), and plan for and grow the venture (Wiklund and Shepherd, 2003; Coleman, 2007; Segal et al., 2007; Hisrich et al., 2008). Prior entrepreneurship exposure facilitates entrepreneurial action and drive the entrepreneur’s efforts in carrying out new venture organising activities (Hopp and Sonderegger, 2015; Botha, 2020). Thus, prior entrepreneurship exposure and work experience can be essential sources of learning that enable individuals to acquire the necessary entrepreneurial skills and can enhance one’s confidence in acting entrepreneurially (Storey and Greene, 2010; Khoshmaram et al., 2018). According to Storey and Greene (2010), individuals’ assessment of the entrepreneurial talent acquired from work experience can affect the entrepreneurial career choice negatively or positively. Such an assessment can affirm whether or not an individual is adequately prepared for an entrepreneurial journey. These authors report that prior employment experience in a large organisation is less likely to propel an individual to become self-employed (Storey and Greene, 2010). In the assessment of the role of work experience and entrepreneurial experience, Giones and Miralles (2020) observed that work experience has no effect on new venture emergence while entrepreneurial experience significantly influences new venture emergence.

Work experience directly influences success in the entrepreneurs’ efforts to create a new venture (Hisrich et al., 2008; Hopp and Sonderegger, 2015) and increases the likelihood of becoming a nascent entrepreneur (Davidsson and Honig, 2003). However, prior research concerning the effect of work experience on entrepreneurial intention indicates mixed findings. The findings of Aragon-Sanchez et al. (2017) show that work experience is negatively related to entrepreneurial intention, attitude and perceived behavioural control and has no significant effect on subjective norms. Similarly, Malebana and Swanepoel (2015) observed the negative influence of prior work experience on entrepreneurial intention. In addition, other studies found no relationship between work experience and entrepreneurial intention (Shinnar et al., 2009; Walter and Dohse, 2009; Abebe, 2012; Egerová et al., 2017), and perceived behavioural control (Dodescu et al., 2021). These findings contradict those of prior research which have shown that work experience is positively related to entrepreneurial intention (Carr and Sequeira, 2007; Edigbo et al., 2021; Sahinidis et al., 2021), perceived behavioural control (Liñán and Chen, 2009; Entrialgo and Iglesias, 2016; Soria-Barreto et al., 2017) and attitude towards behaviour (García-Rodríguez et al., 2015). Additionally, a study by König (2016) indicates that work experience positively affects attitude towards entrepreneurship and perceived behavioural control and is negatively related to subjective norms. Based on the literature reviewed above, it is hypothesised that:

*H1*: Prior work experience has a statistically significant relationship with entrepreneurial intention.

*H2*: Prior work experience has a statistically significant relationship with attitude towards behaviour.

*H3*: Prior work experience has a statistically significant relationship with subjective norms.

*H4*: Prior work experience has a statistically significant relationship with perceived behavioural control.

Exposure to entrepreneurship can take place through entrepreneurship education, role models, prior start-up experience, by working in a small enterprise or through one’s own current engagement in entrepreneurial activities (Krueger, 1993; Gird and Bagraim, 2008; Malebana, 2012; Zapkau et al., 2015; Tarek, 2016; Malebana and Zindiye, 2017; Botha, 2020; Gulzar and Fayaz, 2021; Muchabaiwa and Msimango-Galawe, 2021). While there is some evidence that prior entrepreneurship exposure has a positive relationship with entrepreneurial intention (Cieślik and Van Stel, 2017; Gulzar and Fayaz, 2021; Muchabaiwa and Msimango-Galawe, 2021), previous research concerning the role of prior entrepreneurship exposure in the formation of entrepreneurial intention offers mixed results. For instance, Tarek (2016) reports that current business ownership, prior start-up experience and entrepreneurial family background have no significant relationship with entrepreneurial intention. Individuals who had prior start-up experience are more likely to display strong entrepreneurial intentions, positive attitude towards entrepreneurship and high confidence in their capability to start a business (Rambe and Ndofirepi, 2017). The effect of prior entrepreneurship exposure on the antecedents of entrepreneurial intention depends on the breadth and perceived quality or positiveness of experiences (Krueger, 1993; Peterman and Kennedy, 2003; Zapkau et al., 2015). Experiencing positive outcomes and success from one’s own engagement in entrepreneurial tasks or observing others succeed and realising positive outcomes from the same behaviour can stimulate entrepreneurial intention and have a positive effect on the antecedents of entrepreneurial intention and vice versa (Bandura, 1986; Naffziger et al., 1994; Marques et al., 2012; Ajzen and Sheikh, 2013; Zhang et al., 2014; Zapkau et al., 2015; Cardoso et al., 2018). Thus, higher breadth and positive experiences enhance perceived feasibility of entrepreneurship (Krueger, 1993). Peterman and Kennedy (2003) observed that positive entrepreneurial experiences increase the desirability of entrepreneurship while Zapkau et al. (2015) report the positive effect of perceived quality of entrepreneurship exposure on subjective norms and attitude.

According to Davidsson and Honig (2003), the likelihood of becoming a nascent entrepreneur is higher among individuals with prior start-up experience than those without it. Aloulou (2017) found that prior entrepreneurial experience is negatively related to entrepreneurial intention, attitude towards behaviour, perceived behavioural control and subjective norms. On the contrary, prior entrepreneurial experience has been found to have a positive effect on perceived behavioural control (Dodescu et al., 2021) and attitude towards entrepreneurship (Basu and Virick, 2008; García-Rodríguez et al., 2015), and subjective norms (König, 2016). It has been found that having self-employment experience has a positive effect on entrepreneurial intention while having self-employed parents or relatives has no influence on entrepreneurial intention (Gird and Bagraim, 2008). A study by Malebana and Zindiye (2017) found that prior start-up experience, having friends who run a business, and knowing someone who is an entrepreneur have a positive effect on entrepreneurial intention. Contrary to these findings, the study of Zhang et al. (2019) revealed that prior entrepreneurship exposure is significantly related to subjective norms but not with entrepreneurial intention, attitude towards entrepreneurship and perceived behavioural control. Self-employment experience is also positively related to subjective norms (Liñán and Chen, 2009). Different types of entrepreneurial role models have varying effects on entrepreneurial intention and the antecedents of intention (Malebana, 2016). However, there are some studies which indicate the positive effects of an entrepreneurial family background or having parental role model on entrepreneurial intention (Carr and Sequeira, 2007; Basu and Virick, 2008; Egerová et al., 2017; Edigbo et al., 2021). Additionally, having parent entrepreneurial role models positively affects attitude towards entrepreneurship (Liñán and Chen, 2009), subjective norms and perceived behavioural control (Basu and Virick, 2008; Walter and Dohse, 2009; Entrialgo and Iglesias, 2016; Palmer et al., 2021). Therefore, it is hypothesised that:

*H5*: Prior entrepreneurship exposure has a statistically significant relationship with entrepreneurial intention.

*H6*: Prior entrepreneurship exposure has a statistically significant relationship with attitude towards behaviour.

*H7*: Prior entrepreneurship exposure has a statistically significant relationship with subjective norms.

*H8*: Prior entrepreneurship exposure has a statistically significant relationship with perceived behavioural control.

**Figure 1:**
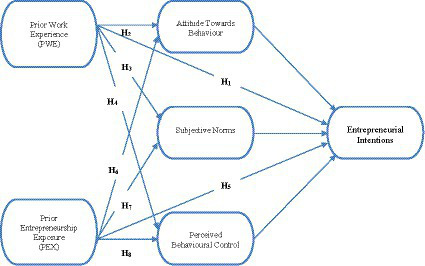
Conceptual model.

The proposed research model, illustrated in Figure 1 below is based on Ajzen’s (1991) theory of planned behaviour, but has been modified to account for the impact of prior entrepreneurship exposure and prior work experience in stimulating entrepreneurial intentions. The model depicts the variables that were used in this research as well as their relationships. The mediating variables were subjective norms, attitude towards behaviour, and perceived behavioural control, while the dependent variable was entrepreneurial intention. Based on the proposed model, the two independent variables, prior work experience and prior entrepreneurship exposure, have an effect on entrepreneurial intention, which is mediated by subjective norms, attitude towards behaviour, and perceived behavioural control.

## Materials and methods

### Sample and procedure

The study population comprised of 1,003 first-, second- and third-year entrepreneurship diploma students enrolled at the TUT in the year 2020. Out of the targeted population, the online survey questionnaire was completed by a convenience sample of 301 students who were willing to participate in the study. This resulted in a 30% response rate. While the sample is not representative, it is however not surprising as online surveys have low response rates (Evans and Mathur, 2005; Nayak and Nayaran, 2019). Table 1 summarises the profile of respondents. Slightly above 49% of the respondents were male while 44.9% were female and 6% did not disclose their gender. In terms of age, 31.2% of the respondents were between the age of 18–21 years old while 42.9% were between 21 and 23 years old and 25.9% were 24 - above. In relations to their academic level, 31.2% were first year students, 34.2% were second year students and 34.6% were third year students.

**Table 1:** Demographic profile of respondents.

Variables	Description	Frequency	Percentage
Gender	Male	148	49.2
	Female	135	44.9
	Not disclosed	18	6
	Total	**301**	**100**
Age	18–20	94	31.2
	21–23	129	42.9
	24 – Above	78	25.9
	Total	**301**	**100**
Entrepreneurship education level of study	First year	94	31.2
	Second year	103	34.2
	Third year	104	34.6
	Total	**301**	**100**

This study was conducted based on a cross-sectional, quantitative approach in which data were collected using an online survey questionnaire. Since the study targeted a large number of students, a quantitative approach that allowed for data collection using a survey questionnaire was appropriate (Saunders et al., 2016). The chosen research methods are also considered appropriate because the results of extensive literature reviews that have been conducted by Liñán and Fayolle (2015) and Lortie and Castogiovanni (2015) indicate that entrepreneurial intention research has been predominantly undertaken using cross-sectional, quantitative research designs, and were conducted using surveys based on structured questionnaires. The data were collected after receiving ethical approval from the Tshwane University of Technology (TUT) Research Ethics Committee. The data collection process began when the researcher sought and was granted permission to disseminate the online survey questionnaire *via* WhatsApp Messenger utilizing a Google Forms survey hyperlink. An entrepreneurship educator at the TUT assisted the researcher by providing contact information for class representatives, allowing the researcher access to three WhatsApp Messenger groups made up entirely of entrepreneurship diploma students studying at TUT and encouraged students to participate in the study.

### Data collection and measures

The data was gathered using a structured online survey questionnaire based on previously validated EI questionnaire (Liñán and Chen, 2009; Malebana, 2012). All questions were adopted from prior EI studies (Liñán and Chen, 2009; Malebana, 2012) with no adjustments. The main reason for adopting measures from these studies is that they have been validated in previous research (Rauch and Hulsink, 2015; Tarek, 2016; Miralles et al., 2017; Mahmoud et al., 2020; Al-Qadasi et al., 2021; Dodescu et al., 2021; Ndofirepi, 2022) and therefore the reliability of the questionnaire and validity of results will be enhanced. Entrepreneurial intention, attitude towards behaviour, perceived behavioural control and subjective norms were measured using a five-point Likert scale (1 = strongly disagree, 5 = strongly agree). Entrepreneurial intention was the dependent variable while attitude towards behaviour, perceived behavioural control and subjective norms were mediators. Attitude towards behaviour, as shown in Table 2, is comprised of six items and had Cronbach’s alpha coefficient of 0.884. Nine items measured perceived behavioural control with Cronbach’s alpha coefficient of 0.904. Entrepreneurial intention consisted of five items with Cronbach’s alpha coefficient of 0.892, while subjective norms is comprised of six items and had Cronbach’s alpha coefficient of 0.889. Prior work experience and prior entrepreneurship exposure were independent variables that were measured using a nominal scale (yes or no).

**Table 2:** The measurement model.

Construct	#Items	Factor loading	Cronbach’s Alpha	CR	AVE
Attitude towards behaviour	ATB1	0.793	0.884	0.912	0.634
	ATB2	0.846			
	ATB3	0.744			
	ATB4	0.848			
	ATB5	0.809			
	ATB6	0.729			
Entrepreneurial intentions	EI1	0.708	0.892	0.921	0.702
	EI2	0.848			
	EI3	0.880			
	EI4	0.874			
	EI5	0.867			
Prior Entrepreneurship Exposure	PEX1	0.641	0.712	0.805	0.511
	PEX2	0.641			
	PEX3	0.715			
	PEX4	0.844			
Perceived behavioural control	PBC1	0.699	0.904	0.921	0.568
	PBC2	0.784			
	PBC3	0.813			
	PBC4	0.716			
	PBC5	0.836			
	PBC6	0.588			
	PBC7	0.794			
	PBC8	0.804			
	PBC9	0.716			
Prior work experience	PWE1	1.000	1.000	1.000	1.000
Subjective norms	SN1	0.837	0.889	0.914	0.640
	SN2	0.782			
	SN3	0.790			
	SN4	0.830			
	SN5	0.750			
	SN6	0.809			

## Results

### Analytical strategy

SmartPLS 4 (Ringle et al., 2022) and Microsoft Excel were used to analyse the data. The sample demographics profile was created using descriptive statistics, and the relationship between the independent variable, mediating variables, and dependent variables was tested using SmartPLS 4. In accordance with Hair et al. (2017) and Sarstedt et al. (2021), we conducted the measurement model assessment and the structural model assessment.

### Measurement model evaluation

SmartPLS 4 was employed to examine the research’s measurement model. Cronbach’s alpha, composite reliability, average variance extracted, convergent validity, and discriminant validity were all used to evaluate the measurement model. Cronbach Alpha values >0.7 are required for constructs to be regarded internally consistent (Hair et al., 2017). However, Churchill (1979) indicated that a Cronbach Alpha scores of between 0.6 and 0.7 are generally considered satisfactory, whereas scores of 0.8 or higher indicate very high consistency. Table 2 demonstrates that all variables had Cronbach Alpha score ranging from 0.68 to 0.92, indicating that all six constructs are internally consistent. Composite reliability values for the measurement model should be more than 0.8 for the constructs to be internally consistent (Hair et al., 2017). Overall, all variables included in this study had composite reliability values more than 0.8. This indicates that these six variables are internally consistent, as indicated by the fact that all of these values range between 0.81 and 0.94. A metrics for establishing convergent validity is average variance extracted (Ravand and Baghaei, 2016). At least 0.5 is required to achieve adequate convergent validity for a concept (Fornell and Larcker, 1981). The average variance extracted values for all of the variables in this study were >0.5, showing good discriminant validity. The square root of the AVE value for each construct should be greater than the squared correlation of the indicators for any other construct in order for discriminant validity to be achieved (Fornell and Larcker, 1981; Hair et al., 2017). The discriminant validity has been accomplished, as shown in Table 3, and the square root of the AVE is greater than the inter-construct correlations (Fornell and Larcker, 1981).

**Table 3:** Discriminant validity (Fornell-Larcker criterion).

Construct	ATB	EI	PEX	PBC	PWE	SN
Attitude towards behaviour	0.796					
Entrepreneurial intentions	0.781	0.838				
Prior entrepreneurship exposure	−0.308	−0.337	0.715			
Perceived behavioural control	0.665	0.660	−0.248	0.754		
Prior work experience	−0.126	−0.110	0.437	−0.154	1.000	
Subjective norms	0.404	0.407	−0.187	0.363	0.021	0.800

Hair et al. (2017) suggest the heterotrait-monotrait ratio (HTMT) as a better alternative and reliable criterion compared to the Fornell-Larcker criterion in assessing discriminant validity. To achieve discrimant validity, HTMT values should not exceed the 0.90 threshold. As shown in Table 4, HTMT values for the constucts were below the required threshold, suggesting that the constructs were distinct and discriminant validity was established (Hair et al., 2017; Sarstedt et al., 2021).

**Table 4:** Heterotrait-monotrait ratio (HTMT) - matrix.

Construct	ATB	EI	PBC	PEX	PWE	SN
Attitude towards behaviour (ATB)						
Entrepreneurial intentions (EI)	0.870					
Perceived behavioural control (PBC)	0.736	0.721				
Prior entrepreneurship exposure (PEX)	0.348	0.368	0.256			
Prior work experience (PWE)	0.135	0.116	0.156	0.478		
Subjective norms (SN)	0.430	0.437	0.394	0.204	0.029	

### Structural model evaluation

**Figure 2:**
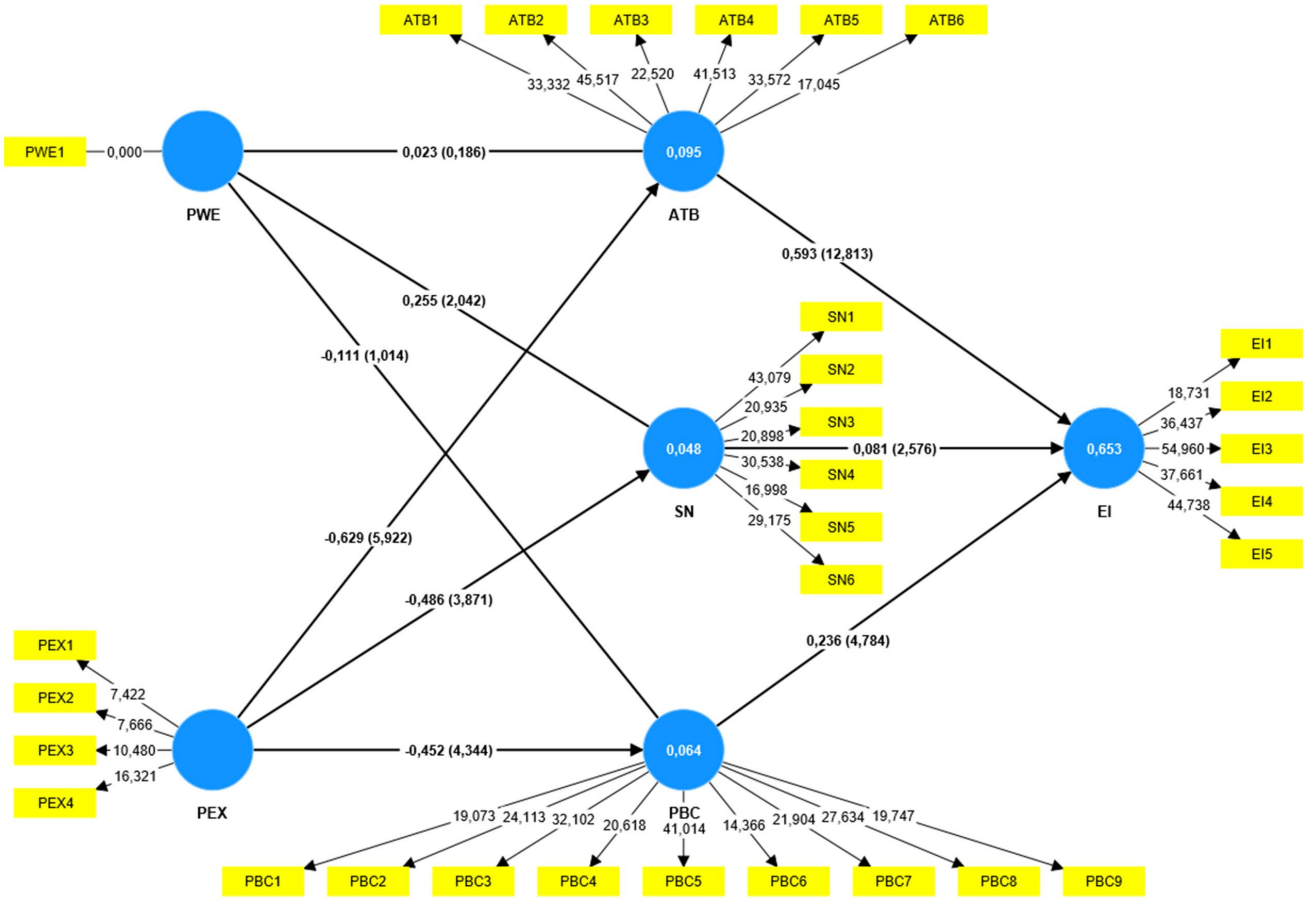
Structural model.

After evaluating the measurement model’s validity and reliability, the proposed structural model is examined (Figure 2). Evaluation of the structural model focuses on the coefficient of determination or the level of *R*^2^ values (Hair et al., 2017). According to Chuan and Penyelidikan (2006), the *R*^2^ of endogenous latent variables should be >0.26 for a decent model. *R*^2^ values of 0.75, 0.50, and 0.25 for the dependent variables are considered significant, moderate, and weak, respectively, by Hair et al. (2017). This study’s cumulative effect of factors on the endogenous latent variable EI is 0.653, showing that the variables’ total impact is positive. The link between latent constructs was evaluated and the conceptual model was validated using a structural model evaluation (Wong, 2013; Hair et al., 2017). Following the evaluation of the measurement model, the current study looked at the structural model by running a bootstrap on 5,000 replicates to see how significant the path coefficient was (Ringle et al., 2015; Hair et al., 2017). Figure 2 illustrates the full estimates of the structural equation model, together with the mediating variables.

### Assessment of collinearity issues

Hair et al. (2017) assert that estimation of weights and their statistical significance can be impacted by high levels of collinearity. According to Hair et al. (2017), VIF values should be less than the threshold value of 5. However, it is suggested that VIF values below 3 give assurance that multicollinearity is unlikely to become a problem (Hair et al., 2019, 2020). So the presence of collinearity issues was assessed in this study and the variance inflation factors (VIF) of indicators as shown in Table 5 met the stated criteria as they are all below the threshold. The VIF values of predictor constructs ranged between 1.218 and 1.897. This means that there are no collinearity issues that could have a negative effect on the results.

**Table 5:** Collinearity statistics (VIF).

Construct	ATB	EI	PBC	PEX	PWE	SN
Attitude towards behaviour		1.897				
Entrepreneurial intentions						
Perceived behavioural control		1.829				
Prior entrepreneurship exposure	1.237		1.237			1.237
Prior work experience	1.237		1.237			1.237
Subjective norms		1.218				

### Direct effects

The path coefficients and their accompanying significance levels were calculated to examine the significance of the structural relationships. The findings in Table 6 indicate that prior work experience has a positive and statistically significant relationship with subjective norms (*β* = 0.255, *p* < 0.05). However, no statistically significant relationship was found between perceived behavioural control, attitude towards behaviour, entrepreneurial intention and prior work experience. This means that as prior work experience increases, subjective norms is predicted to increase. These findings provide support for *H3*, while *H1*, *H2* and *H4* are rejected.

**Table 6:** Path coefficients for direct effects.

Path	Path coefficient	Mean	Standard deviation	*t*-statistics	*p-*value
PWE - > ATB	0.023	0.031	0.121	0.186	0.852
PWE - > EI	0.008	0.016	0.097	0.080	0.936
PWE - > PBC	−0.111	−0.105	0.109	1.014	0.311
PWE - > SN	0.255	0.265	0.125	2.042	0.041
PEX - > ATB	−0.629	−0.648	0.106	5.922	0.000
PEX - > EI	−0.519	−0.539	0.086	6.026	0.000
PEX - > PBC	−0.452	−0.473	0.104	4.344	0.000
PEX - > SN	−0.486	−0.508	0.126	3.871	0.000
ATB - > EI	0.593	0.590	0.046	12.813	0.000
PBC - > EI	0.236	0.238	0.049	4.784	0.000
SN - > EI	0.081	0.082	0.031	2.576	0.010

The findings indicate that prior entrepreneurship exposure has a negative but statistically significant relationship with entrepreneurial intention (*β* = −0.519, *p* < 0.001), subjective norms (*β* = −0.486, *p* < 0.001), attitude towards behaviour (*β* = −0.629, *p* < 0.001) and perceived behavioural control (*β* = −0.452, *p* < 0.001). This means that as students’ prior entrepreneurship exposure increases, the entrepreneurial intention, perceived behavioural control, subjective norms and attitude towards behaviour are predicted to decrease. These findings provide support for *H5*, *H6*, *H7*, and *H8*.

Furthermore, the results show that perceived behavioural control (*β* = 0.236, *p* < 0.001), subjective norms (*β* = 0.081, *p* < 0.05) and attitude towards behaviour (*β* = 0.593, *p* < 0.001) have a positive and statistically significant relationship with entrepreneurial intention. The findings provide full support for the theory of planned behaviour as all the three predictors are significantly related to entrepreneurial intention.

Table 7 summarises the results of the hypotheses test and structural model assessment. The summary of the findings of this study suggests that prior work experience had a positive but statistically significant relationship only with subjective norms (*β* = 0.255, *p* < 0.05) while prior entrepreneurship exposure had a negative but statistically significant relationship with entrepreneurial intention (*β* = −0.519, *p* < 0.001), subjective norms (*β* = −0.486, *p* < 0.001), perceived behavioural control (*β* = −0.452, *p* < 0.001) and attitude towards behaviour (*β* = −0.629, *p* < 0.001). As a result, hypotheses *H3*, *H5*, *H6*, *H7*, and *H8* were accepted at *p* < 0.05 significance level, and *H1*, *H2*, and *H4* were rejected at a significance level of *p* > 0.05.

**Table 7:** Summary of structural model assessment.

Hypothesis	Hypothesised path	Path coefficient	Mean	Standard deviation	*t*-statistics	*p*-value	Level of significance
H_1_	PWE - > EI	0.008	0.016	0.097	0.080	0.936	n.s
H_2_	PWE - > ATB	0.023	0.031	0.121	0.186	0.852	n.s
H_3_	PWE - > SN	0.255	0.265	0.125	2.042	0.041	*
H_4_	PWE - > PBC	−0.111	−0.105	0.109	1.014	0.311	n.s
H_5_	PEX - > EI	−0.519	−0.539	0.086	6.026	0.000	***
H_6_	PEX - > ATB	−0.629	−0.648	0.106	5.922	0.000	***
H_7_	PEX - > SN	−0.486	−0.508	0.126	3.871	0.000	***
H_8_	PEX - > PBC	−0.452	−0.473	0.104	4.344	0.000	***

*p < 0.05, **p < 0.01, ***p < 0.001.

### Mediation effects of the antecedents of entrepreneurial intention

Bootstrapping of the sampling distribution was performed in order to test the indirect effects, as suggested by Hair et al. (2017). This technique is considered having higher levels of statistical power compared to the Sobel test and provides more accurate results. The findings in Table 8 show that perceived behavioural control (*β* = −0.107, *p* < 0.01), and attitude towards behaviour (*β* = −0.373, *p* < 0.001) mediate the negative relationship between prior entrepreneurship exposure and entrepreneurial intention. Subjective norms was insignificant in mediating the relationship between prior entrepreneurship exposure and entrepreneurial intention. However, this is a partial mediation because prior entrepreneurship exposure has a significant negative direct effect on entrepreneurial intention (Hair et al., 2017). No significant indirect effect was found between prior work experience and entrepreneurial intention.

**Table 8:** Mediation effects.

Path	Path coefficient	Mean	Standard deviation	*t*-statistics	*p*-value	Mediation
PEX - > ATB - > EI	−0.373	−0.383	0.071	5.289	0.000	***
PEX - > SN - > EI	−0.039	−0.043	0.021	1.869	0.062	n.s
PEX - > PBC - > EI	−0.107	−0.113	0.036	2.929	0.003	**
PWE - > ATB - > EI	0.013	0.019	0.072	0.187	0.852	n.s
PWE - > SN - > EI	0.021	00.022	0.014	1.518	0.129	n.s
PWE - > PBC - > EI	−0.026	−0.025	0.027	0.962	0.336	n.s

*p < 0.05, **p < 0.01, ***p < 0.001.

## Discussion

The purpose of this study was to investigate the effects of prior work experience and prior entrepreneurship exposure on entrepreneurial intentions of students at a university of technology in South Africa. The findings revealed that prior work experience had a statistically significant relationship with subjective norms and an insignificant relationship with perceived behavioural control, attitude towards behaviour and entrepreneurial intention. These findings indicate that having work experience only increases the perceived social pressure to engage in entrepreneurship but has no effect on perceived behavioural control, attitude towards behaviour and entrepreneurial intention. The results corroborate prior research that showed that work experience has no significant relationship with entrepreneurial intention (Shinnar et al., 2009; Walter and Dohse, 2009; Abebe, 2012; Egerová et al., 2017), and perceived behavioural control (Dodescu et al., 2021). However, the results contradict those of Aragon-Sanchez et al. (2017) which found no significant effect of prior work experience on subjective norms. Additionally, the findings are in contrast with previous research that reported a significant relationship between prior work experience and entrepreneurial intention (Carr and Sequeira, 2007; Edigbo et al., 2021; Sahinidis et al., 2021), perceived behavioural control (Liñán and Chen, 2009; Entrialgo and Iglesias, 2016; Soria-Barreto et al., 2017) and attitude towards behaviour (García-Rodríguez et al., 2015; König, 2016). Unlike perceived behavioural control and entrepreneurial intention, subjective norms has not been found to be associated with entrepreneurial behaviour and previous research results for its relationship with entrepreneurial intention are mixed, suggesting that prior work experience is unlikely to drive individuals into the entrepreneurial career option.

Moreover, the results showed that prior entrepreneurship exposure is negatively related to attitude towards behaviour, entrepreneurial intention, perceived behavioural control and subjective norms. This means that when prior entrepreneurship exposure of the respondents increased their attitude towards behaviour, entrepreneurial intention, perceived behavioural control and subjective norms decreased. The results indicate that prior entrepreneurship exposure did not make a positive contribution in enhancing entrepreneurial intention and the antecedents of intention among the respondents. These results corroborate Aloulou (2017) who reported a negative relationship between prior entrepreneurial experience and entrepreneurial intention, and its three antecedents. Similarly, Malebana (2014b) alluded that prior start-up experience and entrepreneurial family background are negatively related to entrepreneurial intention. Findings contradict previous research that positively associated prior entrepreneurship exposure to entrepreneurial intentions (Carr and Sequeira, 2007; Basu and Virick, 2008; Egerová et al., 2017; Malebana and Zindiye, 2017; Edigbo et al., 2021), attitude towards entrepreneurship (Liñán and Chen, 2009) and perceived behavioural control (Basu and Virick, 2008; Walter and Dohse, 2009; Entrialgo and Iglesias, 2016; Palmer et al., 2021) and subjective norms (Liñán and Chen, 2009; König, 2016; Zhang et al., 2019). The negative relationship between prior entrepreneurship exposure and entrepreneurial intention and its antecedents among the respondents could possibly be interpreted in the light of the quality of their prior entrepreneurship exposure. The respondents’ entrepreneurial intentions and the antecedents thereof are unlikely to be strengthened when they experience failure and negative outcomes from performing entrepreneurial tasks or when they observe their own role models experiencing negative outcomes or failure (Bandura, 1986; Naffziger et al., 1994; Ajzen and Sheikh, 2013; Zhang et al., 2014; Zapkau et al., 2015; Cardoso et al., 2018).

Furthermore, the findings revealed that the entrepreneurial intention of students at the TUT was predicted by subjective norms, attitude towards behaviour and perceived behavioural control. These findings corroborate those of previous research that reported the full support of the theory of planned behaviour in predicting entrepreneurial intention (Gird and Bagraim, 2008; Walter and Dohse, 2009; Kibler et al., 2014; Malebana, 2014b; Kautonen et al., 2015; Zapkau et al., 2015; König, 2016; Miralles et al., 2016; Aragon-Sanchez et al., 2017; Mwiya et al., 2017; Soria-Barreto et al., 2017; Tarek, 2017; Mothibi and Malebana, 2019; Zhang et al., 2019; Dodescu et al., 2021; Saleh and Manjunath, 2022; Sampene et al., 2022). However, the results contradict those of prior research which discovered an insignificant relationship between entrepreneurial intention and attitude towards behaviour (Ndofirepi, 2022), perceived behavioural control (Shah and Soomro, 2017; Sharaf et al., 2018; Mahmoud et al., 2020; Al-Qadasi et al., 2021), and subjective norms (Malebana and Swanepoel, 2015; Ndofirepi and Rambe, 2017; Sharaf et al., 2018; Mahmoud et al., 2020; Al-Qadasi et al., 2021; Sahinidis et al., 2021; Akter and Iqbal, 2022; Bazan, 2022).

### Theoretical implications

The results of our study have advanced the theory of planned behaviour by showing that this theory is a valuable model for testing the effects of prior entrepreneurship exposure on entrepreneurial intention and its antecedents. By testing the effects of prior entrepreneurship exposure using the theory of planned behaviour we offer a complete view of how prior entrepreneurship exposure shapes entrepreneurial intention. However, this study measured prior work experience and prior entrepreneurship exposure using yes or no types of questions. As a result, these measures could not cater for the nature and quality of experiences. Testing the effects of the nature and quality of experiences would help in advancing the theory, especially with regards to shedding light on the types of experiences that impact the formation of entrepreneurial intention. There is relatively limited research on the role of prior work experience in the formation of entrepreneurial intention, therefore, further research is required.

### Practical and policy implications

The findings of this study have several implications that can be beneficial for both policymakers and entrepreneurship educators. Entrepreneurial activity is an intentionally planned activity that depends on perceived attractiveness of the entrepreneurial career option and perceived ability to act entrepreneurially. Extant literature shows that prior entrepreneurship exposure can play a crucial role in equipping individuals with entrepreneurial knowledge to identify and exploit opportunities in the market. The findings in this study raise doubts on the quality of entrepreneurial experiences to which students were exposed. The negative relationship between prior entrepreneurship exposure and attitude towards behaviour, entrepreneurial intention, perceived behavioural control and subjective norms indicate that entrepreneurship experiences did not have a positive influence on the antecedents of entrepreneurial intention and neither did they stimulate entrepreneurial intention. Policymakers should provide those already engaged in entrepreneurial behaviour with the different types of support they need so they could be successful in their ventures. Doing so will strengthen entrepreneurs’ intention and the antecedents of entrepreneurial intention. The more these entrepreneurs succeed and achieve positive outcomes in their ventures, those observing them would also feel encouraged to engage in entrepreneurial behaviour with the confidence that they too will succeed and realise similar outcomes. Policymakers should partner with the media to showcase successful entrepreneurs. That will create a positive image of the entrepreneurial career option and entrepreneurs.

Entrepreneurship education is one of the sources of entrepreneurship exposure, which according to the literature shapes the formation of entrepreneurial intention and also impacts the antecedents of entrepreneurial intention. In view of the results of this study, entrepreneurship educators should create learning environments that expose students to meaningful and positive entrepreneurial experiences. These educators should adopt experiential teaching methods which allow students to learn by doing and experiment with entrepreneurship by starting ventures as part of their learning. Doing so will assist students in gaining entrepreneurial knowledge. Entrepreneurship educators should use teaching techniques that include flipped classroom, discussions, scenarios, case studies, collaboration learning, projects and problem-based learning to foster entrepreneurship in the classroom. There is a need to value both failures and successes in the learning process as they all contribute to an individual’s growth and development. In the same vein, exposing students to both failure and successful entrepreneurial role models would provide students with a realistic picture of what it takes to thrive in entrepreneurship.

## Limitations and directions for future research

This study is not without limitations, some possible limitations can be revealed as guidelines for future research. Firstly, this study was cross-sectional in nature, and therefore no causality can be inferred. Longitudinal research could help to accurately depict the effects of prior work experience and prior entrepreneurship exposure on changes observed in the theory of planned behaviour components and entrepreneurial intention over time. Secondly, the findings cannot be generalised beyond the scope of this study because the focus was purely on entrepreneurship students at the TUT. The low response rate prevents generalisation of the results to the target population. Thirdly, the limitation of quantitative studies is that they are carried out in unnatural environments and therefore, do not provide rich data that captures feelings, thoughts and behaviours of the participants that are associated with qualitative studies. Future research should consider investigating the effects of prior work experience and prior entrepreneurship exposure at universities in South Africa offering entrepreneurship and other disciplines to validate the findings of this study. There is a need to examine the effects of the nature and quality of entrepreneurship exposure on entrepreneurial intention.

## Conclusion

This study contributes to the advancement of the theory of planned behaviour by testing the effects of prior work experience and prior entrepreneurship exposure on entrepreneurial intention and the antecedents of entrepreneurial intention in the South African context. Contrary to prior research, this study has shown that the effect of prior entrepreneurship exposure varies from one population to another. Such effects could also be dependent on the nature and quality of entrepreneurial experiences. The study also tested the mediation effects of the antecedents of entrepreneurial intention in the relationship between prior work experience and prior entrepreneurship exposure and entrepreneurial intention. The results indicate that prior work experience plays no role in formation of entrepreneurial intention, while prior entrepreneurship exposure negatively impacts entrepreneurial intention, attitude towards behaviour, perceived behavioural control and subjective norms. These findings suggest that students who had work experience were not engaged in work responsibilities that equipped them with the skills they could use to start their own businesses and neither did such responsibilities increase the desirability for the entrepreneurial career option among students. These results suggest need for interventions that could expose students to positive entrepreneurial experiences. Exposing students to positive entrepreneurial experiences would make the entrepreneurial career attractive and would enhance perceived capability for starting a business. Individuals are more likely to feel encouraged to start a business when they see successful entrepreneurs who could serve as their role models. Similarly, entrepreneurship education that provides opportunities for students to experiment with their ideas will increase the attractiveness of the entrepreneurial career and perceived capability to start a business, especially when students realise success in their experimental efforts. In line with majority of prior research, this study has affirmed the predictive validity of the theory of planned behaviour.

## Data availability statement

The raw data supporting the conclusions of this article will be made available by the authors, without undue reservation.

## Author contributions

SM contributed to the conception and data analysis of the study. MM wrote the introduction, literature review, edited the results, wrote the discussion, implications, and conclusion. All authors contributed to the article and approved the submitted version.

## Conflict of interest

The authors declare that the research was conducted in the absence of any commercial or financial relationships that could be construed as a potential conflict of interest.

## Publisher’s note

All claims expressed in this article are solely those of the authors and do not necessarily represent those of their affiliated organizations, or those of the publisher, the editors and the reviewers. Any product that may be evaluated in this article, or claim that may be made by its manufacturer, is not guaranteed or endorsed by the publisher.
